# In vitro cytotoxic and apoptotic effects of boric acid on endometrial adenocarcinoma cell lines (HEC-1B and Ischikawa)

**DOI:** 10.1007/s12032-025-02625-4

**Published:** 2025-02-19

**Authors:** Nazlı Çil, Elif Önder, Ayşe Nur Damar, Seyedmahdi Tabatabaei, Ümit Çabuş, Gülçin Abban Mete

**Affiliations:** 1 Department of Histology and Embryology, Faculty of Medicine, Pamukkale University, Pamukkale, Denizli, Turkey; 2 Department of Obstetrics and Gynecology, Faculty of Medicine, Pamukkale University, Pamukkale, Denizli, Turkey

**Keywords:** Boric acid, HEC-1B, Ishikawa, Endometrial adenocarcinoma, Apoptosis

## Abstract

Endometrial carcinoma, the most common malignancy of the female genital tract, remains challenging to treat despite early-stage dominance. Surgical interventions and irradiation are insufficient for advanced endometrial cancer. Our aim was to investigate to explore the in vitro cytotoxicity and apoptotic effects of boric acid (BA) on endometrial adenocarcinoma cell lines (Ishikawa and HEC-1B cell lines), providing experimental evidence for the potential application of boric acid as an anticancer drug. Time- and dose-dependent cell viability was determined with the XTT cell proliferation test. Differences in mRNA levels were determined by RT-PCR using cDNAs and SYBR green assay. Colony formation and the effect of BA on wound healing were evaluated. Immunocytochemistry and TUNEL tests were performed to evaluate apoptosis. BA increased the expression of Caspase 3 and Bax in HEC-1B and Ischikawa cell lines. It was determined that BA significantly decreased the number of colonies in both cell lines (*p *< 0.05). In HEC-1B and Ishikawa cell lines, there was an increase in cell migration in the control group at 16 and 24 h. The apoptotic index was higher in the BA group, although it was not statistically significant. According to immunohistochemistry results, Caspase 3 and Bax expression in HEC-1B and Ishikawa cell lines were statistically increased in BA group. The expression of Bcl-2 was decreased statistically with BA treatment in both cell lines (*p* = 0.0001). BA treatment inhibited cell migration and colony formation, which are important for carcinogenesis, in endometrial adenocarcinoma cell lines. This inhibition was shown to occur through the apoptotic pathway.

## Introduction

Endometrial adenocarcinoma is a common gynecological cancer that ranks as fifth cancer worldwide [1]. It usually occurs after the menopause. However, 2–14% of patients are women under the age of 40 who want to preserve their fertility [2]. More than 380,000 new cases of endometrial carcinoma were reported worldwide. In 2018, with endometrial adenocarcinoma accounting for approximately 85% of these cases [3, 4]. The abnormal growth of endometrial epithelial cells primarily leads to endometrial adenocarcinoma [5]. Chemotherapy remains one of the most potent therapies for endometrial malignancies. However, like all malignancies, chemotherapy’s side effects persist, affecting patients’ quality of life. Consequently, research has been ongoing for years to discover novel, safer treatments for malignant cells while minimizing harm to healthy ones.

Boron, a rare element, plays biological roles in animals, plants, yeasts, and bacteria and its evolutionary significance dates back to prebiotic ages [6]. Dietary boron, an essential component of human nutrition, is primarily sourced from plant-based boron compounds and boric acid (BA) dissolved in water [7, 8]. Boron has beneficial effects on biological functions in humans and animals. These include reproduction, growth, calcium metabolism, bone formation, energy metabolism, immunity, and brain function [9]. The median total boron intake from dietary food is approximately 1.0–1.5 mg/day for adults [10]. Boric acid is the most common form of boron found in the human body [9]. Boric acid is swiftly absorbed from the gastrointestinal tract after dietary intake, enters the bloodstream, and participates in numerous physiological and biochemical processes due to its antioxidant or anticancer properties [11–13]. Boron deficiency causes several effects, including stunted growth, weakened bones, altered steroid hormone concentrations, impaired neuronal function, and altered immune response (9). Several epidemiological studies indicate that exposure to BA is associated with a decreased incidence of prostate cancer, cervical cancer, and lung cancer [14–16]. Experimental studies have demonstrated that BA reduces cell proliferation and/or triggers apoptosis in prostate, melanoma, and breast cancer cell lines [17–22]. Research has shown that BA possesses unique cytotoxic effects on cancer cells. In an experiment involving nude mice, which were injected with androgen-sensitive LNCaP prostate cancer cells, treatment with BA led to decreased tumor growth and suppression of prostate-specific antigen enzymatic activity [17]. In cell culture or animal studies, researchers have observed that boron compounds demonstrate anti-proliferative effects [9, 23]. Although the cellular effects of these compounds in relation to cancer remain unclear, they continue to captivate researchers due to their multifaceted impact on cancer treatment and other diseases [24].

Understanding the cellular alterations induced by BA is pivotal for forthcoming anticancer investigations. These studies will unveil the potential therapeutic benefits of BA and provide guidance for further research in this domain. In our current study, we focused on comprehensively assessing the cellular changes in endometrial adenocarcinoma cell lines resulting from treatment with different concentrations of BA. We aimed to evaluate the effect of BA on cell migration, colony formation, and the apoptotic pathway in endometrial cell lines.

## Materials and methods

### Reagents

DMEM-F12 was purchased from Sigma (Sigma-Aldrich, St. Louis, MO, USA), RPMI was purchased from Serox (Serox GmbH, Mannheim, Germany), penicillin–streptomycin, and trypsin were purchased from Gibco (Sigma-Aldrich, St. Louis, MO, USA), phosphate-buffered saline (PBS) and fetal bovine serum (FBS) were obtained from Capricorn (Capricorn Scientific, Ebsdorfergrund, Germany), primary antibodies were from Affinity (Affinity Biosciences, USA), BA was obtained from Eti Maden (Eti Maden İşletmeleri, Ankara, Türkiye), secondary kit was from Thermo (Thermo Fisher Scientific, Massachusetts, USA), TUNEL kit was obtained from Elabscience (Elabscience Bionovation, United States), XTT kit was from Biological Industries (Sartorius AG, Göttingen, Germany), and cDNA synthesis kit and syber green A.B.T. Retrieved were from (Atlas Biotechnology, Ankara, Turkey).

### Cell lines and cell culture

Human Ishikawa cells (American Tissue Culture Collection (ATCC®, Manassas, VA, USA)) were cultured in RPMI 1640 containing 10% FBS, 1% l-glutamine, and 1% penicillin–streptomycin. Human HEC-1B cells (American Tissue Culture Collection (ATCC®, Manassas, VA, USA)) were cultured in DMEM-F12 containing 10% FBS, 1% l-glutamine, and 1% penicillin–streptomycin. Cells were passaged every 2–3 days depending on their density and monitored in an incubator at 37 °C, 95% humidity, and 5% CO_2_.

### Cell viability assay

Time- and dose-dependent cell viability were determined with the XTT cell proliferation test, and the dose at which 50% of the cells were alive was found. Cell viability test was performed using the XTT kit (BI Cell Proliferation Kit XTT-based Colorimetric Assay, LOT: 2,046,899). Ishikawa cells were seeded with RPMI, and HEC-1B cells were seeded in DMEM-F12 in 96-well plates at 15,000 cells per well. After the cells were incubated for 24 h, the medium was removed. BA was dissolved in sterile distilled water. The concentration-dependent cytotoxic effect was determined. For HEC-1B cell line 25, 50, 100, 200, 300, 400, and 500 mM, and for Ishikawa cell line, doses of 50 μM, 100 μM, 200 μM, 400 μM, 800 μM, 1 mM, 5 mM, 10 mM, 20 mM, and 40 mM of BA were prepared and administered to the cells [25, 26]. Some cells were not given BA and were considered as the control group. Each dose group was studied in triplicate. Doses were applied for 24, 48, and 72 h to analyze the time-dependent effect. After 24, 48, and 72 h of incubation, the absorbance values of the groups were read with the ELISA device in the wavelength reference range of 450–630 nm. Percent cell viability was calculated by dividing the measured optical density value in each well by the control optical density value and multiplying by one hundred to determine the IC 50 ratio.

### RNA isolation, cDNA synthesis, and real-time PCR (RT-PCR)

HEC-1B and Ishikawa cells were added to six-well plates as control and dose groups in triplicate, with 400,000 cells in each well, and allowed to adhere. IC50 dose was applied to the dose group and kept incubated throughout the day. The medium was then removed from the cells and 500-µl Trizol was applied to each group. Total RNA was isolated. cDNA was synthesized according to the cDNA synthesis Kit protocol. Differences in mRNA levels were determined by RT-PCR using SYBR green assay. GAPDH was used as the housekeeping gene. The primer sequences for Caspase 3, Bax, and Bcl-2 are shown in Table 1.

**Table 1:** Real-Time PCR forward and reverse primer sequences

Gene names	Primer sequence	Accession numbers
GAPDH	F: GTCTCCTCTGACTTCAACAGCG R: ACCACCCTGTTGCTGTAGCCAA	NM_002046
Caspase 3	F: GGAAGCGAATCAATGGACTCTGG R: CATCGACATCTGTACCAGACC	XM_54350958.1
BAX	F: TCAGGATGCGTCCACCAAGAAG R: TGTGTCCACGGCGGCAATCATC	NM_004324
Bcl-2	F: ATCGCCCTGTGGATGACTGAGT R: GCCAGGAGAAATCAAACAGAGGC	NM_000633

### Colony formation assay

The cells (1 × 10^3^) were seeded in 6-well plates and they were incubated for 24 h. Control and dose groups were incubated at 37 °C for 14 days, following the medium. After colonies formed, the cells were fixed with methanol at − 20 ͦ °C and then stained with crystal violet. The experiments were repeated 3 times. Colony numbers of groups were evaluated statistically.

### Wound healing

The cells (1 × 10^6^) were seeded in each well of 6-well plates. After the cells adhered to the surface in 100% confluency, the medium was removed, a wound was created at the base of the cells using a 200-µl pipette tip. It was washed with PBS 3 times to clean the cells debris. BA IC50 dose was added to the groups. Media were added to the groups. The wound closure was photographed at time 0, 16, and 24 h. The experiments were repeated 3 times. It was analyzed with the ImageJ program.

### Apoptosis determination with TUNEL

HEC-1B and Ishikawa cells were planted in an 8-well chamber slide with 30,000 cells in each well. After the cells adhered, BA IC50 dose was added to the experimental groups, and medium was added to the groups and incubated. When the IC50 dose day was completed, the cells’ medium was removed and the cells were fixed with methanol at − 20 °C. Fixed cells were stained according to the Elabscience (FITC) kit protocol. DAPI was used as counterstaining. The staining was examined under an immunofluorescence microscope. TUNEL-positive cells stain green with FITC and TUNEL-negative cells stain blue with DAPI. The apoptotic index (AI) was calculated by counting 10 random areas under the microscope at 20 × magnification [27].

## Immunocytochemistry

The cells were seeded in 8-well chamber slides (3 × 10^4^). After waiting 1 day for the transplanted cells to become adhesive, the media was withdrawn and fixed with methanol at − 20 °C. Caspase 3, Bax, and Bcl-2 primary antibodies diluted 1:100 were applied to the cells. The cells were treated with streptavidin peroxidase enzyme and DAB chromogen according to the protocol of the secondary kit. Cells that were counterstained with hematoxylin and examined for cytoplasm and nucleus staining. Cells without staining were scored as 0 (negative), weakly stained as + 1, moderately + 2, and strongly stained cells as + 3 [28, 29].

### Statistical analysis

The analysis of the data was quantified by computer program using the 2^−ΔΔCt^ method. The groups were compared using the Volcano Plot analysis featured in the “RT^2^ Profiler; PCR Array Data Analysis” program. The groups were statistically evaluated by the “Student *t* test” analysis. In addition, parametric and non-parametric analyses of all groups were evaluated with SPSS 17.0 statistical analysis program. In the analysis of the data in the study, the Shapiro–Wilk test was used to determine whether the group distributions were normal (*p* < 0.05 indicates statistical significance).

## Results

### Cell viability assay

**Fig. 1:**
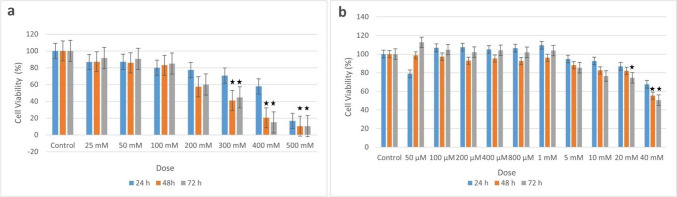
Effect of boric acid on the viability of HEC1B vs Ishikawa endometrial adenocarcinoma cell lines. The cells were treated with boric acid at different doses and times and their proliferation was assessed by XTT assay. *p < 0.05 vs Control group. For HEC1B (**a**), the IC50 dose at 72 h was 377.75 mM; for Ishikawa (**b**), the IC50 dose at 72 h was 28.45 mM

According to the XTT result, the IC50 dose of BA was determined as 377.75 mM for HEC-1B at 72 h when compared to the control group. For Ishikawa cells, the BA dose was found to be 28.45 mM at 72 h compared to the control group. In the cell viability test results at 24 and 48 h, cell proliferation did not fall below 50% (Fig. 1).

### Real-time PCR

It was determined that BA increased the expression of Caspase 3 by 3.54 times and Bax expression by 2.67 times in the HEC-1B cell line. However, there was no statistically significant difference compared to the control group. It was observed that BA increased Caspase 3 expression by 2.98 times and Bax expression by 1.84 times in the Ischikawa cell line (*p* > 0.05) (Table 2).

**Table 2:** The mRNA expression changes of genes in Endometrial Adenocarcinoma Cell Lines (HEC1B-Ishikawa) treated with groups compared with the control group cells

Gene names	HEC1B BA		Ishikawa BA	
	Fold regulation	*p* value	Fold regulation	*p* value
GAPDH	1.00	nan	1.00	nan
Caspase 3	3.54	0.31	2.98	0.13
BAX	2.67	0.08	1.84	0.14
Bcl-2	1.95	0.29	1.31	0.68

Data were obtained by Real-time polymerase chain reaction (RT-PCR) assay via 2^−ΔΔCt^ method in RT2 Profiles PCR Array Data Analysis online program

*BA* Boric acid, *p* < 0.05 statistically significant

### Colony formation assay

**Fig. 2:**
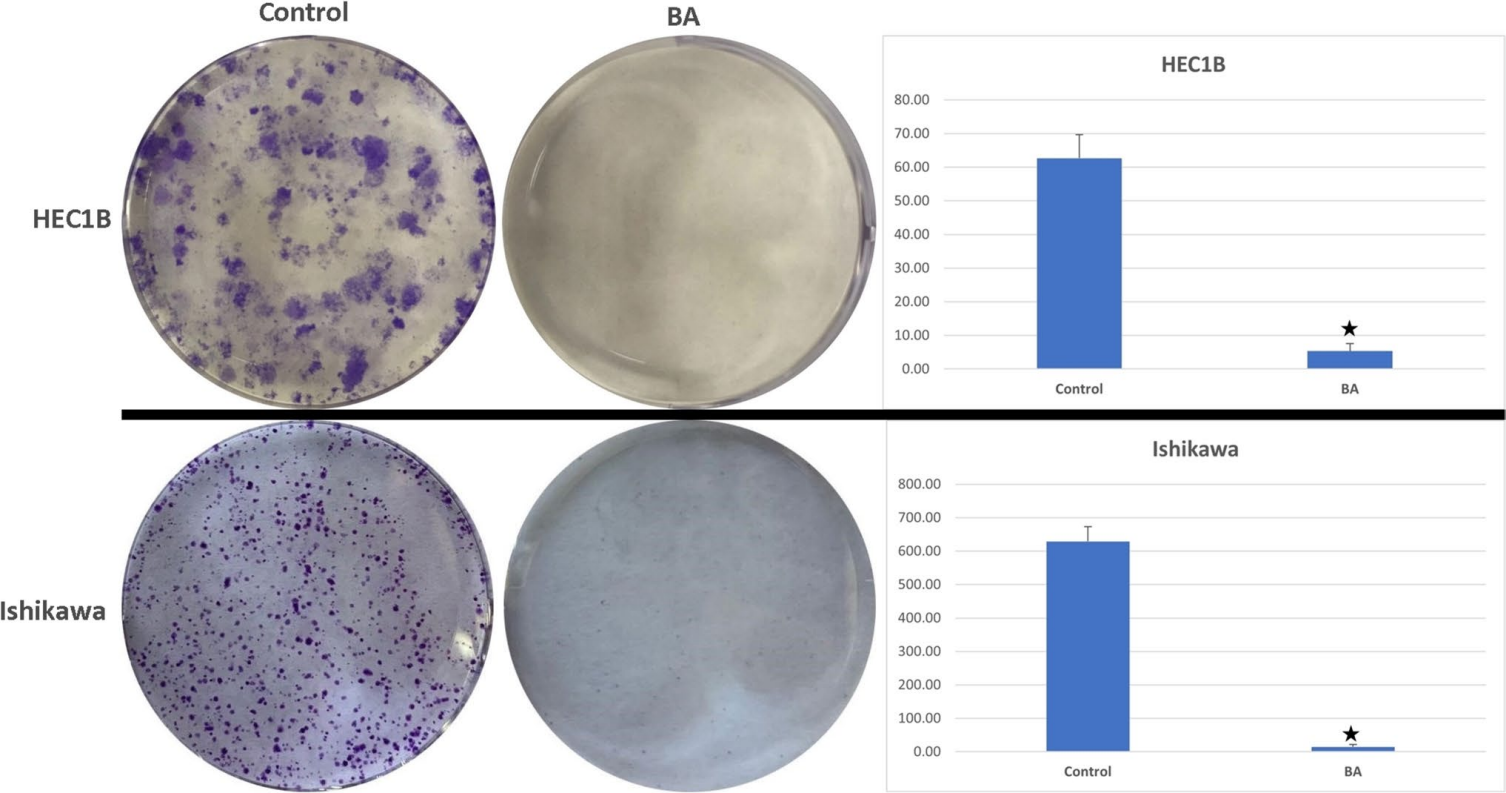
Effects of boric acid on colony formation of HEC1B and Ishikawa cells after 14 days. Colonies were stained with crystal violet. For both cells, control groups formed significantly more colonies than the boric acid-treated group (*p < 0.05 vs Control group) (BA: Boric acid)

At the end of 14 days, the average colony number of HEC-1B cells was 62.66 ± 6.94 in the control group and 5.33 ± 2.17 in the BA group. Colony formation in the control group of Ischikawa cell line was 629.00 ± 44.26, while it was 14.66 ± 6.72 in the BA group. As a result of the statistical analysis between the groups, it was determined that BA decreased the number of colonies in both cell lines statistically significantly (*p* < 0.05) (Fig. 2).

### Wound healing assay

**Fig. 3:**
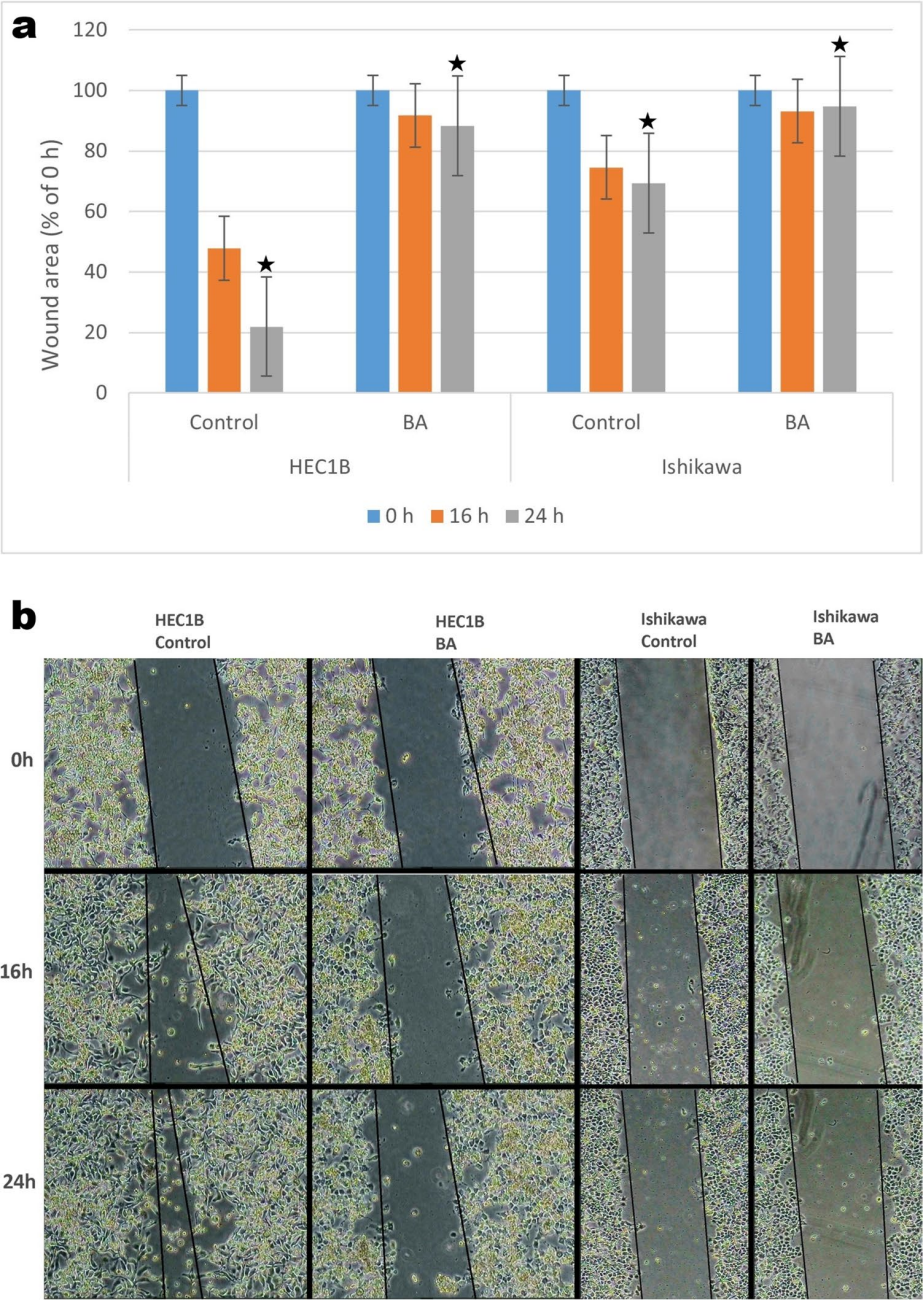
Effect of boric acid on migration in HEC1B and Ishikawa cells by wound healing assay. Boric acid significantly reduced migration in HEC1B and Ishikawa cell lines compared to control at the end of 24 h (p* < 0.05) (BA: Boric acid)

Our results showed that in HEC-1B and Ishikawa cell lines, there was an increase in cell migration in the control group at 16 and 24 h. The migration of HEC-1B and Ishikawa cells at 24 h was found to be statistically higher. In the BA-treated group, cell migration decreased at the 16th h. This decrease was statistically significant, especially at 24 h. At the same time, we found that cell migration in HEC-1B and Ishikawa cells was less in BA group compared to control group at 16 h. At 24 h, cell migration was statistically significantly decreased in both cell lines compared to BA group control group (*p* < 0.005) (Fig. 3).

### TUNEL assay

**Fig. 4:**
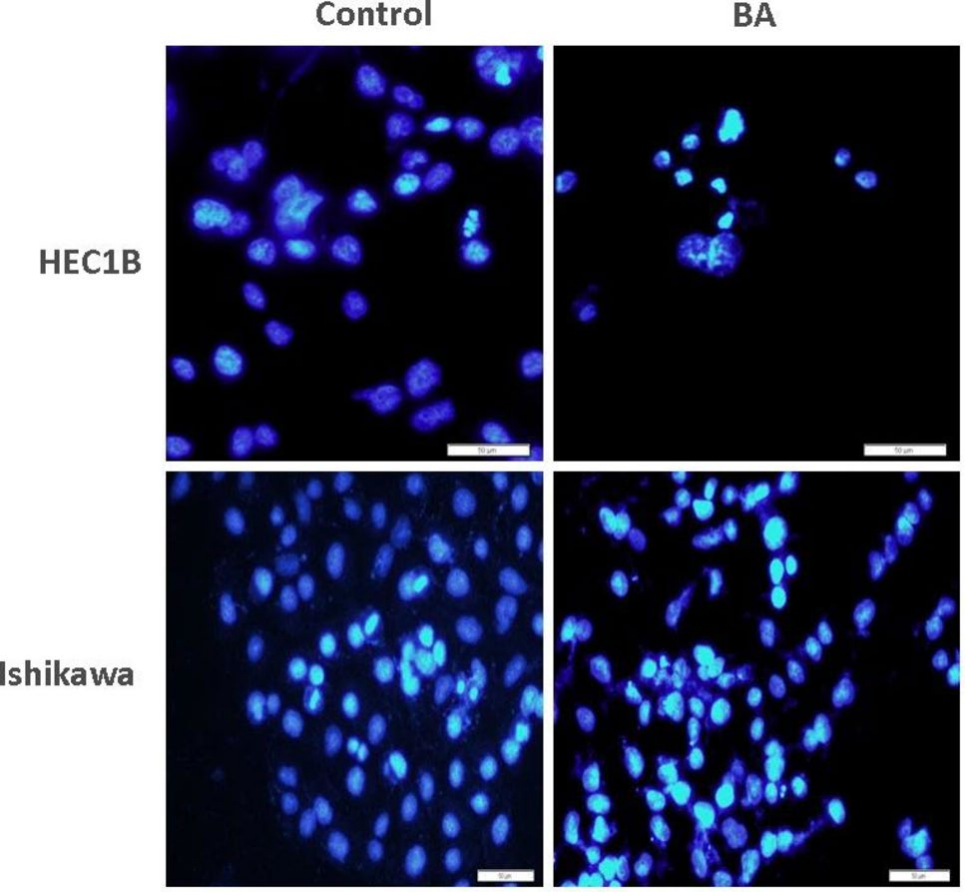
Evaluation of the apoptotic effects of boric acid in HEC1B and Ishikawa cells by TUNEL staining. In HEC1B cell line, boric acid increased the number of TUNEL-positive cells compared to the control group (*p* < 0.05). In Ishikawa cell line, although there was no difference statistically, the number of TUNEL-positive cells increased (*p* > 0.05) (BA: Boric acid)

In the TUNEL staining performed after 72 h of exposure to BA, it was determined that the majority of HEC-1B cells were shed and most of the remaining cells were stained TUNEL-positive compared to the control group (*p* < 0.05). When AI was calculated, it was found to be higher in the BA group, although it was not statistically significant. BA to the Ishikawa cell line resulted in an increase of AI compared to the control group. However, this difference was not statistically significant (*p* > 0.05) (Fig. 4).

### Immunocytochemistry

**Fig. 5:**
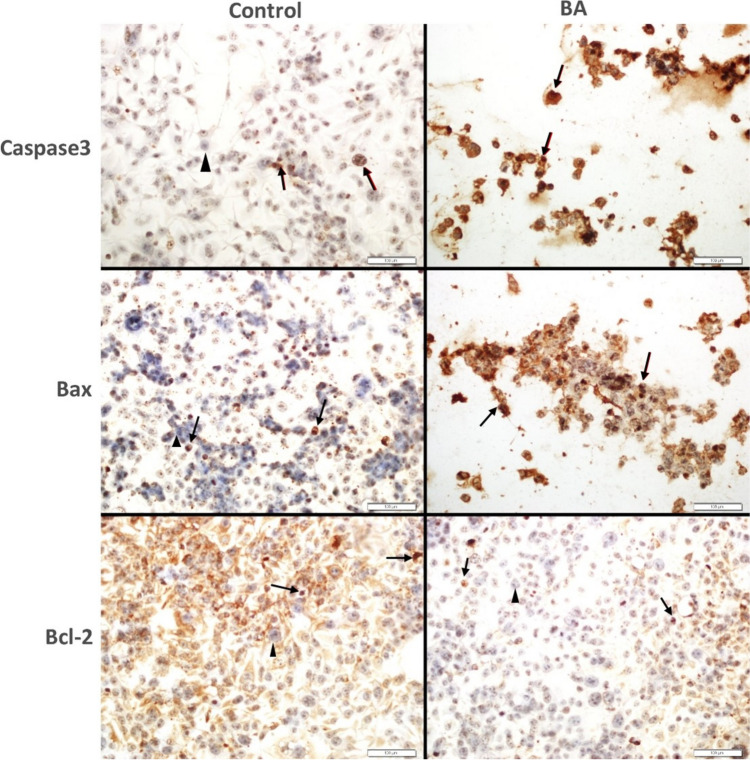
Immunohistochemical studies in 2D culture. In HEC1B cell line, Caspase 3 and BAX expression significantly increased, while Bcl-2 expression significantly decreased (*p* = 0.0001). Arrow: positive expression, arrowhead: negative expression (BA: Boric acid)

**Fig. 6:**
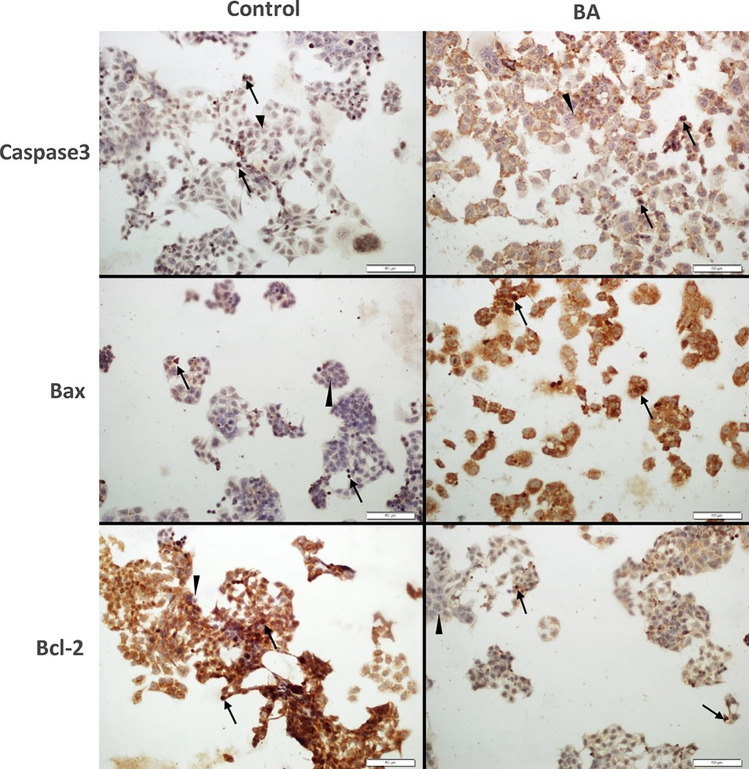
Immunohistochemical studies in 2D culture. In Ishikawa cell line, Caspase 3 and BAX expression significantly increased, while Bcl-2 expression significantly decreased (*p* = 0.0001) Arrow: positive expression, arrowhead: negative expression (BA: Boric acid)

The expression of Caspase 3 and Bax was significantly increased by BA treatment in HEC-1B and Ishikawa cell lines. (*p* = 0.0001). The expression of Bcl-2 was observed to significantly decreased by BA treatment in both cell lines (*p* = 0.0001) (Figs. 5, 6).

## Discussion

BA demonstrated several beneficial effects in endometrial cancer cells. Specifically, it reduced cell viability, induced apoptosis, activated oxidative stress, and suppressed inflammatory responses. Notably, even at its IC50 concentration (40 mM after 24 h of incubation), BA did not harm normal fibroblasts. These favorable properties suggest that BA may be a valuable therapeutic option for inhibiting the development and progression of endometrial cancer [30]. Sevimli et al. investigated the effect of BA in suppressing cell proliferation via the TNF signaling pathway-mediated apoptosis in the human colon cancer cell line. Their findings suggest that BA holds promise as a potential candidate for anticancer treatment in colon cancer [26]. Canturk et al. examined the impact of BA and sodium tetraborate on both acute leukemia cell line and healthy human lymphocytes [25]. Studies have reported that BA inhibits the growth of LNCaP prostate tumors in nude mice, resulting in a 64% reduction in the likelihood of developing prostate cancer compared to men who consumed the least amount of boron. Treatment of nude mice, which were injected with androgen-sensitive LNCaP prostate cancer cells, using BA resulted in a 25–38% decrease in tumor growth, along with an 88% reduction in plasma prostate-specific antigen levels [31]. In culture, BA has been shown to dose dependently inhibit the proliferation of LNCaP and the androgen-independent prostate cancer cell lines DU-145 and PC-3 [26, 31]. DU-145 cells were exposed to varying doses of BA (ranging from 0 to 1000 μM) for 1, 2, and 7 days. By day 7, there was a decrease in protein expression levels of cyclins A, B1, C, D1, and E, as well as the phosphorylated form of the MAPK signaler MEK at concentrations of 500 and 1000 μM. p53 expression remained stable at higher concentrations, whereas p21 decreased after 7 days of exposure [31]. In our study, the dose of BA that killed 50% of the cells was determined as 377.75 mM for HEC-1B and 28.45 mM for Ishikawa. It was noticeable that the doses were higher than the BA dose used in prostate cancer and colon cancer.

Certain boron compounds exhibit antitumor properties across various cancer cell lines [25]. At physiological concentrations, boron acts as a reversible competitive inhibitor of cyclic ADP ribose, which is the endogenous agonist of the ryanodine receptor calcium [32]. BA induces a dose-dependent decrease in cyclins A-E and MAPK proteins, implying their role in inhibiting cell proliferation. Cells treated with BA exhibit diminished adhesion, migration, and invasion capabilities, accompanied by changes in F-actin that suggest a decrease in metastatic potential [31]. The treatment with BA influenced cellular motility, caused morphological alterations and impacted endoplasmic reticulum stress in prostate cancer cell lines [31, 33]. Boric acid dose dependently reduced the proliferation of DU-145 human prostate cancer cells and inhibited cell migration and invasion [13]. Another studies have indicated that boron compounds within the range of 1–1000 μM exhibit inhibitive effects on the metastatic and proliferative characteristics of cancer cells [9]. In our study, inverted, fluorescent, and light microscope images were evaluated to detect morphological changes in the cells. According to our results, it was determined that BA reduced the adhesion ability of Ishikawa cells, especially in HEC-1B cells. In our study, BA inhibited cell colonization and migration in both endometrium adenocarcinoma cell lines.

Acerbo and colleagues investigated the effect of different doses of BA (ranging from 5 to 50 mM) on the viability of human melanoma cells over various time intervals (1–10 days). BA decreased cell viability and induced apoptosis in a dose- and time-dependent manner [19]. Bradke and colleagues compared the impact of BA and phenylboronic acid (PBA) on the migration of prostate and breast cancer cell lines, as well as non-tumorogenic cells from the same tissues. Within the initial 24 h, the compounds did not affect cell adhesion or viability, but they did cause changes in cell morphology. However, after eight days, both compounds significantly reduced cancer cell viability. They observed that BA inhibited cell migration and reduced cell viability after eight days in DU-145, PC-3 prostate, and ZR-75-1 breast cell lines, especially at high doses [26].

The effects of BA on the SW-480 human colon adenocarcinoma cell line have been investigated both 2D and 3D culture systems. Furthermore, a qRT-PCR investigation was conducted to detect alterations in the expression of pivotal genes associated with apoptosis. The results suggest that BA suppresses cell proliferation and induces apoptosis in both 2D and 3D culture environments. It has been found that the observed apoptotic process is associated with the TNF signaling pathway [26].

The potential mechanisms of protection within the human body of BA are reduction of Caspase 3 gen and improvement in proinsulin production, calcium metabolism regulation, reduction of ROS concentration in pancreas, decreasing of carbohydrates and lipids plasma concentration, and immunomodulation [13].

In the MDAH-2774 ovarian cancer cell line, BA upregulated the expression of apoptosis-inducing genes, such as Bax, Bid, Caspase 3, and Caspase 9. It downregulated the expression of anti-apoptotic proteins like Bcl-2 and Bcl-xL. BA reduced the colony-forming, migration, and invasion capacity of MDAH-2774 cells [34]. In our study, consistent with previous studies, BA treatment increased Caspase 3 and Bax statistically significantly in endometrium adenocarcinoma cell lines. TUNEL-positive cells increased with BA treatment, proving that BA activates the apoptotic pathway.

Our most important limitation in this study was the use of BA in high doses. Another limitation of our study was that we could not support our results with 3D culture and animal experiments. Therefore, our future aim is to examine the effects of BA on endometrial adenocarcinoma cells by coating it with a suitable nanoparticle that allows us to apply BA at lower doses and to increase its effect on cancer cells and reduce its damage to normal cells.

## Conclusion

Treatment with BA inhibited cell migration and colony formation in endometrial adenocarcinoma cell lines. This inhibition was shown to occur via the apoptotic pathway. The expression of Caspase 3 and Bax increased, while Bcl-2 expression decreased. Further studies are necessary to demonstrate the efficacy of BA application in the treatment of endometrial adenocarcinoma by 3D culture and animal studies. The findings of this study can provide valuable guidance for future research on the use of BA in treating endometrial adenocarcinoma.

## Data Availability

No datasets were generated or analyzed during the current study.
